# Data mining and analysis of adverse drug events of propofol in the general population and the elderly based on the US Food and Drug Administration Adverse Event Reporting System

**DOI:** 10.1097/MD.0000000000047947

**Published:** 2026-03-06

**Authors:** Qirong Liu, Weijie Ma, Chouqi Hu, Chunyue Fang, Yuanyuan Zhong, Jin Wen

**Affiliations:** a College of Pharmacy, Dali University, Dali, China; b Department of Pharmacy, The Third People’s Hospital of Yunnan, Kunming, China.

**Keywords:** adverse drug events, elderly population, FAERS, propofol, signal mining

## Abstract

This pharmacovigilance study analyzed 8249 adverse event reports for propofol in the general population and 1530 reports in patients aged ≥65 years using the US Food and Drug Administration Adverse Event Reporting System from Q1 2004 to Q1 2025. Employing 3 disproportionality analysis algorithms – reporting odds ratio, proportional reporting ratio, and multi-item gamma Poisson shrinker – we identified 27 and 26 system organ class signals in the general and elderly populations, respectively. Of these, 10 and 8 system organ class signals demonstrated positive disproportionality in the respective groups. Elderly patients exhibited a significantly higher proportion of cardiac disorder reports than the general population, accompanied by markedly stronger signals. Propofol infusion syndrome represented the strongest positive signal across both populations. Notably, acute right ventricular failure emerged as the most prominent cardiac preferred term signal in both groups and appears to be a distinctive adverse reaction unique to propofol. Comparative analysis with sevoflurane (n = 3620) revealed shared cardiovascular effects between the 2 agents; however, propofol demonstrated significantly elevated signals for immune system disorders and metabolic and nutritional disorders. The most frequently reported adverse events in the general population were anaphylactic shock, hypotension, and propofol infusion syndrome, whereas the elderly population most commonly reported anaphylactic shock, hypotension, and cardiac arrest. This study identified important safety signals associated with propofol use and revealed potential age-specific adverse reaction patterns. These findings are exploratory in nature and provide hypothesis-generating evidence; further research is required to confirm these risk associations.

## 1. Introduction

Advances in surgical techniques and perioperative management have progressively lowered the operative threshold, allowing a growing number of geriatric patients to undergo elective or emergency procedures.^[1]^ Age-related decline in respiratory reserve and the higher prevalence of active pulmonary disease markedly increase the risk associated with inhalational anesthetics; consequently, intravenous anesthesia is usually preferred in this population because it offers superior haemodynamic stability and controllability.^[2]^ Propofol was first marketed in the United Kingdom in 1986 and introduced into China a decade later.^[3,4]^ Owing to its rapid onset, short context sensitive half life, and excellent dose–response predictability, propofol has become the intravenous hypnotic of choice for induction and maintenance of general anesthesia as well as for procedural sedation.^[5]^ However, the pharmacokinetics and pharmacodynamics of propofol change significantly with age: decreased systemic clearance and enhanced cerebral sensitivity result in higher blood concentrations at any given dose, predisposing older adults to exaggerated cardiorespiratory depression and other adverse drug events (ADEs).^[6]^ The US Food and Drug Administration Adverse Event Reporting System (FAERS) is a publicly accessible pharmacovigilance repository that captures post-marketing safety reports submitted by healthcare professionals, manufacturers, and consumers. With more than 20 million records, FAERS constitutes a rich data source for signal detection and hypothesis generation regarding drug-related harm.^[7,8]^ Nevertheless, age-stratified safety analyses of propofol remain scarce. The present study therefore, leveraged FAERS data to systematically identify ADE signals associated with propofol in both the general and elderly populations, aiming to delineate age-specific safety profiles and to furnish evidence-based recommendations for the rational use of propofol in geriatric anesthesia.

## 2. Materials and methods

### 2.1. Data extraction and analysis

**Figure 1.**
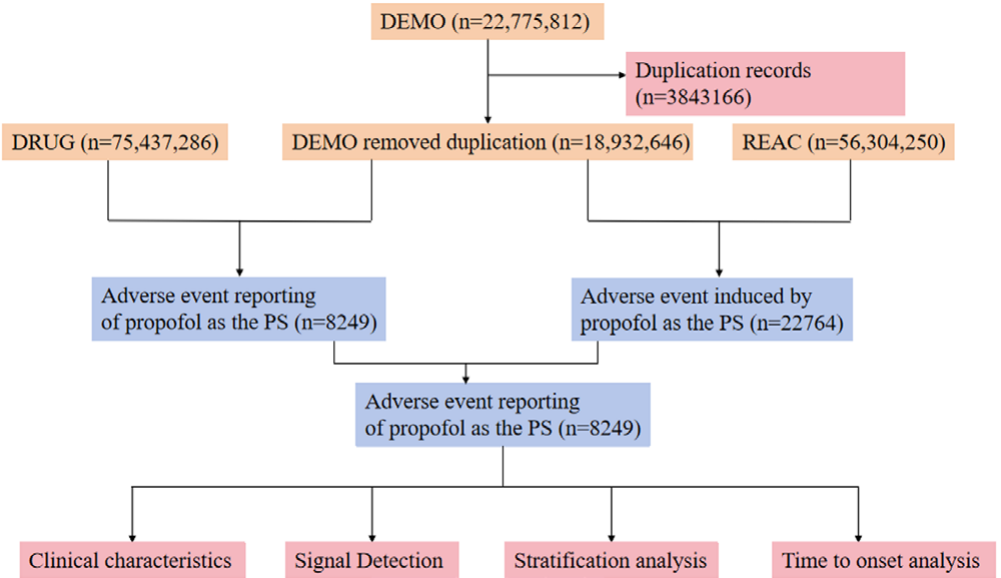
The flow diagram of propofol-related adverse events. DEMO = demographics, DRUG = drug information, PS = primary suspect, REAC = drug reaction information.

ASCII files covering the period 2004 Q1 to 2025 Q1 were downloaded from the FAERS database for both the general and elderly populations exposed to propofol. The files comprised: demographic information (DEMO), drug information (DRUG), adverse events (REAC), patient outcomes (OUTC), report sources (RPSR), therapy start and end dates (THER), and indications (INDI). All files were imported into R 4.4.2 for integration, cleaning, and standardization. The data cleaning rule follows the US Food and Drug Administration (FDA)-recommended duplicate removal procedure: the fields PRIMARYID, CASEID, and FDA_DT from the DEMO table are selected, and the dataset is sorted by CASEID, FDA_DT, and PRIMARYID. For reports with identical CASEID, the record with the largest FDA_DT is kept; if both CASEID and FDA_DT are identical, the record with the largest PRIMARYID is retained. Reports containing misspelled drug names, duplicates, or unrelated adverse events were excluded. Adverse events were mapped to the PTs of MedDRA version 28.1 and grouped by system organ class (SOC). All possible propofol-containing products were identified from FDA-registered sources (Drugs@FDA). Generic and proprietary names of propofol were used to query the “drugname” and “prod_ai” fields in DRUG. Only records in which DRUG.role_cod = “PS” (primary suspect) were retained. After these steps, all propofol-related adverse event reports from 2004 Q1 to 2025 Q1 were extracted from the cleaned dataset. Figure 1 illustrates the entire analysis process. Due to the anonymous coding system employed by FAERS, this study was exempt from institutional review board approval.

### 2.2. Filtering and categorization of signals

Disproportionality analysis based on a 2 × 2 contingency table (Table 1) was employed to identify potential associations between propofol and specific adverse events; this pharmacovigilance technique compares the observed number of reports linking a drug event pair with the number expected under the assumption of independence, or with the background frequency observed for all other drugs in the database, and when the observed count significantly exceeds the expected count a “disproportionality” is declared implying a possible safety signal,^[9]^ using 3 complementary algorithms reporting odds ratio (ROR),^[10]^ proportional reporting ratio (PRR),^[11]^ and multi-item gamma Poisson shrinker^[12]^ with a signal retained only when all 3 metrics simultaneously met their predefined thresholds (Table 2) to minimize false-positive findings.

**Table 1:** 2 × 2 contingency table for disproportionality analysis.

Drug	Target ADEs with drug of interest	ADEs with other drugs	Total
Drug of interest	*a*	*b*	*a* + *b*
Other drug	*c*	*d*	*c* + *d*
Total	*a* + *c*	*b* + *d*	*a* + *b* + *c* + *d*

ADEs = adverse drug events.

**Table 2:** Signal detection method and thresholds.

Method	Formula	Threshold
Reporting odds ratio (ROR)	$ROR=\frac{\left(a/c\right)}{(b/d)}=\frac{ad}{bc}$ $SE\;\;\;\left(ln\;\;\;ROR\right)=\sqrt{\frac{1}{a}+\frac{1}{b}+\frac{1}{c}+\frac{1}{d}}$ $95\%\;\;\;CI=e^{ln(ROR)\pm 1.96}\sqrt{\frac{1}{a}\;\;\;+\frac{1}{b}+\frac{1}{\;\;\;c}+\frac{1}{d}}\;\;\;$	*a* ≥ 3 and 95% CI (lower limit) > 1
Proportional reporting ratio (PRR)	$PRR=\frac{a/(a+b)}{a/(c+d)}$ $SE\;\;\;\left(ln\;\;\;PRR\right)=\sqrt{\frac{1}{a}\;-\frac{1}{a+b}+\frac{1}{\;c}-\frac{1}{c+d}}$ $ln\;(PRR)\pm 1.96\%\sqrt{\frac{1}{a}\;\;\;-\frac{1}{a+b}+\frac{1}{\;\;\;c}-\frac{1}{c+d}}\;\;\;$ $95\%\;\;\;CI=e^{ln(PRR)\pm 1.96\%}\sqrt{\frac{1}{a}\;\;\;-\frac{1}{a+b}+\frac{1}{\;\;\;c}-\frac{1}{c+d}}\;\;\;\;\;\;\;$	*a* ≥ 3 and 95% CI (lower limit) > 1
Multi-item gamma Poisson shrinker (MGPS)	$EBGM=\frac{a(a+b+c+d)}{\left(a+c)+(a+b\right)}$ $95\%\;\;\;CI=e^{ln(PRR)\pm 1.96\%}\sqrt{\frac{1}{a}-\frac{1}{a+b}+\frac{1}{\;\;\;c}-\frac{1}{c+d}}\;\;\;\;\;\;\;$	EBGML > 2 generates a signal, where EBGML denotes the lower limit of the 95% CI of the empirical Bayesian geometric mean (EBGM)

CI = confidence interval, EBGM = empirical Bayesian geometric mean, EBGML = the lower limit of 95% confidence intervals of empirical Bayesian geometric mean, MGPS = multi-item gamma Poisson shrinker, PRR = proportional reporting ratio, ROR = reporting odds ratio, SE = standard error.

### 2.3. Study population classification

Older adults are conventionally defined as individuals aged 65 years or older; however, chronologic age alone has recognized limitations.^[13]^ To facilitate comparisons, the study population was divided into 2 groups: the overall population and the elderly population, defined as those aged ≥65 years.

### 2.4. Comparator drug analysis

To partially address signal specificity, we conducted a supplementary analysis using sevoflurane, an inhaled general anesthetic, as a nonideal comparator. Sevoflurane was selected due to its widespread use in surgical anesthesia, but we acknowledge fundamental differences: route of administration (inhaled vs intravenous), and distinct toxicities (e.g., malignant hyperthermia vs propofol infusion syndrome [PRIS]). We extracted FAERS reports mentioning sevoflurane Q1 2004 to Q1 2025, n = 3620) and applied the same data cleaning pipeline to calculate SOC level ROR values and cardiac disorder PT level ROR values. In the PT level comparative analysis of cardiac disorders, we selected positive PT signals shared by both sevoflurane and propofol for evaluation.

## 3. Results

### 3.1. Clinical characteristics of ADE reports associated with propofol

**Figure 2.**
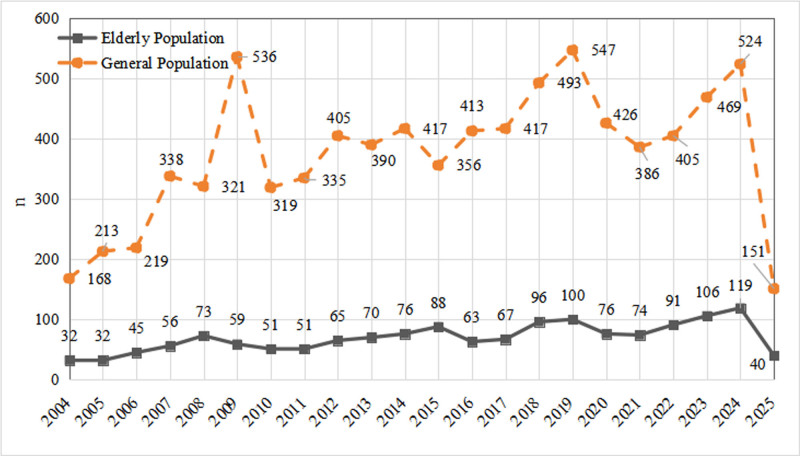
Yearly distribution of adverse event reports for propofol in the general population and the elderly. n = the number of reports.

**Figure 3.**
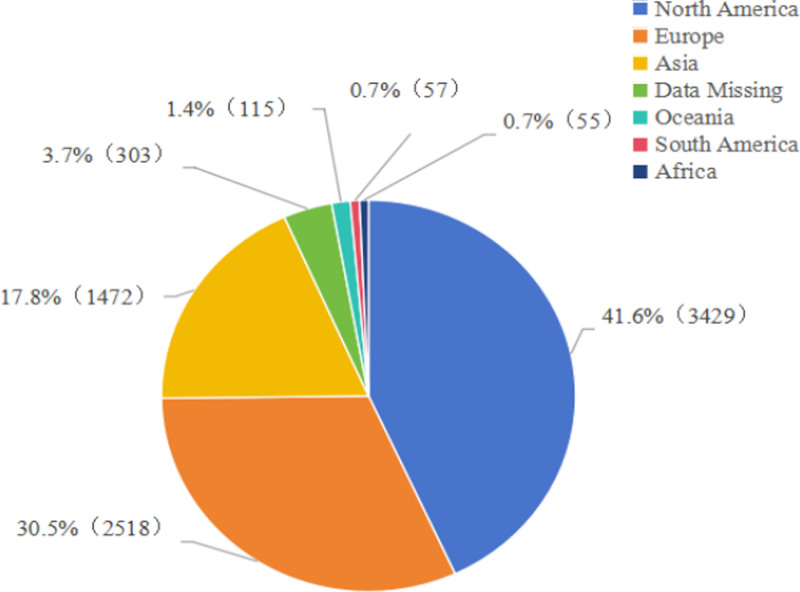
Geographical distribution of all samples.

The clinical characteristics of propofol-related adverse event reports are depicted in Figures 2 and 3 and Table 3. From Q1 2004 to Q1 2025, a total of 8249 adverse events involving propofol were reported in the general population, whereas 1530 events were reported in the elderly. Although annual counts fluctuated markedly in the general population, an overall upward trend was evident; in contrast, reports in the elderly showed a relatively steady rise with a transient dip in 2025 due to incomplete data. Among the general population reports, 3385 cases (46.9%) occurred in males and 3826 (53.1%) in females, indicating a slight female predominance. Conversely, in the elderly subgroup, 792 cases (53.0%) were male and 703 (47.0%) were female, showing a male predominance. The data predominantly came from North America (41.6%) and Europe (30.5%). The largest contributing countries were the United States of America (3129 samples, 37.9% of the total), France (11.5%), Japan (9.9%), the Netherlands (5.7%), and the United Kingdom (5.7%), and were submitted primarily by healthcare professionals pharmacists, physicians, other health specialists, registered nurses, and other healthcare providers. Outcome analysis revealed that the proportions of life-threatening events and hospitalizations were slightly higher in the elderly than in the general population, underscoring the need for heightened safety vigilance when propofol is used in older patients.

**Table 3:** Clinical characteristics of adverse event reports for propofol system organ distribution of adverse event signals for propofol in the general population and the elderly.

Clinical features	N (%)	
	General population	Elderly population
Gender		
Male	3385 (46.9)	792 (53.0)
Female	3826 (53.1)	703 (47.0)
Reporting country		
United States of America	3129 (37.9)	480 (31.4)
France	950 (11.5)	216 (14.1)
Japan	818 (9.9)	264 (17.3)
Netherlands	473 (5.7)	108 (7.1)
United Kingdom	467 (5.7)	85 (5.6)
Other countries	2412 (29.3)	377 (24.5)
Reporter		
Pharmacist	1087 (14.1)	230 (16.0)
Physician	2388 (31.1)	496 (34.6)
Other health professional	2390 (31.1)	396 (27.6)
Consumer	674 (8.8)	120 (8.4)
Lawyer	52 (0.7)	3 (0.2)
Healthcare professional	1067 (13.9)	187 (13.0)
Registered nurse	22 (0.3)	2 (0.1)
Outcome		
Death	992 (10.3)	140 (9.7)
Life-threatening	2077 (21.5)	330 (23.0)
Hospitalization	2449 (25.4)	414 (29.0)
Disability	171 (1.8)	29 (2.0)
Congenital anomaly	15 (0.1)	2 (0.1)
Permanent impairment	277 (2.9)	36 (2.5)
Other serious event	3668 (38.0)	489 (33.9)

Individuals with missing data were excluded from the study inclusion criteria.

### 3.2. Systemic organ distribution of propofol-associated adverse event signals: a comparison between the general and elderly populations

**Figure 4.**
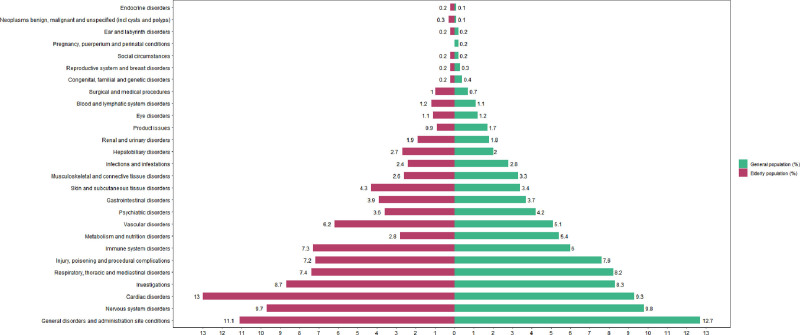
SOC distribution of propofol-related adverse event reports in the general and elderly populations. SOC = system organ class.

In the general population, adverse event signals involved 27 SOCs, while in the elderly population, 26 SOCs were identified (Fig. 4). In the general population, the most frequently affected SOCs were: general disorders and administration site conditions (n = 2922, ROR = 0.67, PRR = 0.71, empirical Bayesian geometric mean [EBGM] = 0.71), nervous system disorders (n = 2262, ROR = 1.14, PRR = 1.13, EBGM = 1.13), cardiac disorders (n = 2138, ROR = 3.66, PRR = 3.43, EBGM = 3.42). In the elderly, the most frequently affected SOCs were: cardiac disorders (n = 553, ROR = 3.91, PRR = 3.54, EBGM = 3.54), general disorders and administration site conditions (n = 472, ROR = 0.66, PRR = 0.69, EBGM = 0.69), nervous system disorders (n = 413, ROR = 1.16, PRR = 1.14, EBGM = 1.14). Compared with the general population, the elderly showed a markedly higher proportion of cardiac disorders and slightly higher proportions of immune system disorders and vascular disorders; the remaining SOCs were largely similar. Signals in the elderly population did not include any events related to pregnancy, puerperium, or perinatal conditions, unlike the general population. Across the 3 disproportionality algorithms, propofol generated positive SOC level signals in 10 SOCs in the general population and 8 in the elderly population. Compared with the overall population, the elderly population showed negative signals for metabolism and nutrition disorders and product issues (Tables 4 and 5).

**Table 4:** Propofol SOC level signal strength in the general population.

SOC	ROR (95% CI)	PRR (χ^2^)	EBGM (EBGML−EBGMU)	N
General disorders and administration site conditions	0.67 (0.64–0.7)	0.71 (419.58)	0.71 (0.68–0.74)	2922
Nervous system disorders*	1.14 (1.1–1.2)	1.13 (37.48)	1.13 (1.08–1.18)	2262
Cardiac disorders*	3.66 (3.5–3.83)	3.43 (3766.62)	3.42 (3.27–3.58)	2138
Investigations*	1.35 (1.29–1.41)	1.32 (160.63)	1.32 (1.26–1.38)	1927
Respiratory, thoracic and mediastinal disorders*	1.75 (1.67–1.83)	1.69 (558.73)	1.69 (1.61–1.77)	1904
Injury, poisoning and procedural complications	0.76 (0.73–0.8)	0.78 (120.36)	0.78 (0.74–0.82)	1748
Immune system disorders*	5.55 (5.26–5.86)	5.29 (4853.91)	5.28 (5–5.57)	1384
Metabolism and nutrition disorders*	2.54 (2.4–2.69)	2.46 (1110.32)	2.46 (2.33–2.61)	1251
Vascular disorders*	2.4 (2.27–2.55)	2.33 (923.21)	2.33 (2.2–2.47)	1188
Psychiatric disorders	0.72 (0.67–0.77)	0.73 (103.51)	0.73 (0.69–0.78)	981
Gastrointestinal disorders	0.4 (0.38–0.43)	0.42 (731.12)	0.42 (0.4–0.45)	857
Skin and subcutaneous tissue disorders	0.61 (0.57–0.65)	0.62 (194.92)	0.62 (0.58–0.67)	792
Musculoskeletal and connective tissue disorders	0.6 (0.56–0.65)	0.62 (192.07)	0.62 (0.57–0.66)	762
Infections and infestations	0.51 (0.47–0.55)	0.52 (299.6)	0.52 (0.48–0.57)	655
Hepatobiliary disorders*	2.17 (1.98–2.38)	2.15 (288.51)	2.15 (1.96–2.36)	465
Renal and urinary disorders	0.96 (0.87–1.05)	0.96 (0.85)	0.96 (0.87–1.05)	414
Product issues*	1.04 (0.94–1.15)	1.04 (0.61)	1.04 (0.94–1.15)	394
Eye disorders	0.56 (0.49–0.63)	0.56 (93.15)	0.56 (0.5–0.63)	268
Blood and lymphatic system disorders	0.64 (0.56–0.72)	0.64 (52.95)	0.64 (0.57–0.72)	257
Surgical and medical procedures	0.48 (0.41–0.56)	0.48 (87.12)	0.48 (0.41–0.57)	157
Congenital, familial and genetic disorders*	1.22 (0.98–1.51)	1.22 (3.32)	1.22 (0.98–1.51)	84
Reproductive system and breast disorders	0.3 (0.23–0.39)	0.3 (95.25)	0.3 (0.23–0.39)	59
Social circumstances	0.52 (0.4–0.68)	0.52 (23.52)	0.52 (0.4–0.68)	54
Pregnancy, puerperium and perinatal conditions	0.46 (0.35–0.62)	0.47 (28.89)	0.47 (0.35–0.62)	47
Ear and labyrinth disorders	0.46 (0.35–0.61)	0.46 (29.76)	0.46 (0.35–0.61)	47
Neoplasms benign, malignant and unspecified (incl cysts and polyps)	0.05 (0.04–0.07)	0.05 (570.35)	0.05 (0.04–0.08)	34
Endocrine disorders	0.55 (0.39–0.77)	0.55 (12.39)	0.55 (0.39–0.77)	33

CI = confidence interval, χ^2^ = chi-squared, EBGM = empirical Bayesian geometric mean, EBGML = the lower limit of 95% confidence intervals of empirical Bayesian geometric mean, EBGMU = the upper limit of 95% confidence intervals of empirical Bayesian geometric mean, PRR = proportional reporting ratio, ROR = reporting odds ratio, SOC = system organ class.

* Denotes a positive signal.

**Table 5:** Propofol SOC level signal strength in the elderly population.

SOC	ROR (95% CI)	PRR (χ^2^)	EBGM (EBGML−EBGMU)	N
Cardiac disorders*	3.91 (3.57–4.27)	3.54 (1044.22)	3.54 (3.24–3.87)	553
General disorders and administration site conditions	0.66 (0.6–0.72)	0.69 (75.84)	0.69 (0.63–0.76)	472
Nervous system disorders*	1.16 (1.05–1.28)	1.14 (8.12)	1.14 (1.03–1.27)	413
Investigations*	1.22 (1.1–1.36)	1.21 (14.02)	1.21 (1.08–1.34)	371
Respiratory, thoracic and mediastinal disorders*	1.25 (1.12–1.41)	1.24 (15.08)	1.24 (1.1–1.39)	315
Immune system disorders*	8.73 (7.78–9.79)	8.18 (1983.23)	8.16 (7.27–9.15)	313
Injury, poisoning and procedural complications	0.77 (0.68–0.86)	0.78 (20.3)	0.78 (0.7–0.88)	309
Vascular disorders*	2.42 (2.14–2.74)	2.34 (208.89)	2.34 (2.06–2.65)	266
Skin and subcutaneous tissue disorders	0.94 (0.81–1.09)	0.94 (0.76)	0.94 (0.81–1.09)	183
Gastrointestinal disorders	0.39 (0.33–0.45)	0.41 (153.85)	0.41 (0.35–0.48)	167
Psychiatric disorders	0.97 (0.83–1.14)	0.97 (0.1)	0.97 (0.83–1.15)	152
Metabolism and nutrition disorders	0.94 (0.78–1.13)	0.94 (0.43)	0.94 (0.78–1.13)	118
Hepatobiliary disorders*	2.71 (2.25–3.26)	2.67 (120.88)	2.67 (2.21–3.21)	115
Musculoskeletal and connective tissue disorders	0.48 (0.4–0.58)	0.5 (60.52)	0.5 (0.41–0.6)	112
Infections and infestations	0.39 (0.32–0.48)	0.41 (92.89)	0.41 (0.33–0.49)	101
Renal and urinary disorders	0.79 (0.63–0.99)	0.79 (4.38)	0.79 (0.64–0.99)	80
Blood and lymphatic system disorders	0.52 (0.39–0.69)	0.53 (21.95)	0.53 (0.4–0.69)	50
Eye disorders	0.53 (0.4–0.7)	0.53 (20.16)	0.53 (0.4–0.71)	48
Surgical and medical procedures	0.69 (0.51–0.94)	0.69 (5.76)	0.69 (0.51–0.94)	41
Product issues	0.77 (0.56–1.07)	0.77 (2.48)	0.77 (0.56–1.07)	37
Neoplasms benign, malignant and unspecified (incl cysts and polyps)	0.1 (0.06–0.18)	0.11 (100.43)	0.11 (0.06–0.18)	13
Reproductive system and breast disorders	0.71 (0.38–1.32)	0.71 (1.16)	0.71 (0.38–1.33)	10
Endocrine disorders	0.75 (0.39–1.45)	0.75 (0.72)	0.75 (0.39–1.45)	9
Congenital, familial and genetic disorders*	2.71 (1.35–5.42)	2.7 (8.59)	2.7 (1.35–5.41)	8
Ear and labyrinth disorders	0.35 (0.17–0.74)	0.35 (8.36)	0.35 (0.17–0.74)	7
Social circumstances	0.57 (0.27–1.2)	0.57 (2.23)	0.57 (0.27–1.2)	7

CI = confidence interval, χ^2^ = chi-squared, EBGM = empirical Bayesian geometric mean, EBGML = the lower limit of 95% confidence intervals of empirical Bayesian geometric mean, EBGMU = the upper limit of 95% confidence intervals of empirical Bayesian geometric mean, PRR = proportional reporting ratio, ROR = reporting odds ratio, SOC = system organ class.

* Denotes a positive signal.

### 3.3. Propofol: signal strength assessment of SOC in the elderly vs the general population

**Figure 5.**
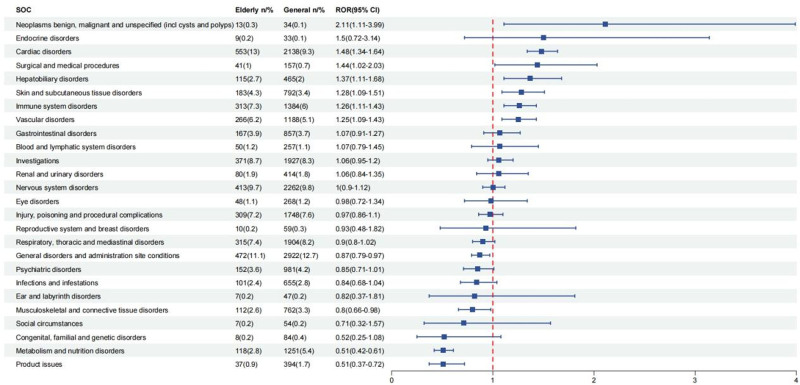
Signal strength assessment of SOCs for propofol in the elderly versus the general population. CI = confidence interval, SOC = system organ class, ROR = reporting odds ratio.

As the IC (information component) cannot be used to compare reporting between subgroups,^[14]^ the relative strength of adverse event signals for each SOC was evaluated by comparing ROR between the elderly and the general population (Fig. 5). Elderly patients exhibited positive signals for the following SOC: neoplasms benign, malignant and unspecified (incl cysts and polyps; n = 13, ROR = 2.11), endocrine disorders (n = 9, ROR = 1.5), and cardiac disorders (n = 553, ROR = 1.48).

### 3.4. Classification of adverse event signals for propofol in the general vs elderly populations

Signal detection analyses identified 412 adverse event signals in the general population and 130 in the elderly. When signals were ranked by reporting frequency and the top 20 were examined, anaphylactic shock and hypotension were the 2 most commonly reported events in both groups (Tables 6 and 7). General population reports more often concerned general disorders and administration site reactions, whereas elderly reports more often involved cardiac disorders. When signals were ranked by strength (ROR), PRIS emerged as a strongly positive signal in both populations (Tables 8 and 9). Among the general population, most high strength signals related to investigations, injuries, poisonings, and procedural complications, including: bispectral index decreased (n = 3, ROR = 874.93, PRR = 874.82, EBGM = 636.51), acute postoperative sialadenitis (n = 4, ROR = 388.87, PRR = 388.81, EBGM = 333.41), airway complications of anesthetics (n = 32, ROR = 379.44, PRR = 378.94, EBGM = 326.13), anesthetic complication pulmonary (n = 10, ROR = 315.38, PRR = 315.25, EBGM = 277.84), premature emergence from anesthesia (n = 4, ROR = 311.1, PRR = 311.05, EBGM = 274.57). Compared with the general population, the elderly showed disproportionately higher risks for several specific events: phaeochromocytoma crisis (n = 4, ROR = 299.51, PRR = 299.24, EBGM = 270.84), acute right ventricular failure (n = 4, ROR = 242.16, PRR = 241.94, EBGM = 223.04), idiopathic interstitial pneumonia (n = 6, ROR = 144.74, PRR = 144.55, EBGM = 137.6), circumoral edema (n = 4, ROR = 137.13, PRR = 137, EBGM = 130.75), and vasoplegic syndrome (n = 15, ROR = 134.55, PRR = 134.09, EBGM = 128.1).

**Table 6:** Top 20 adverse events associated with propofol in the general population, ranked by frequency.

SOC	PT	ROR (95% CI)	PRR (χ^2^)	EBGM (EBGML−EBGMU)	N
Immune system disorders	Anaphylactic shock	88.56 (82.45–95.12)	85.63 (65,055.16)	82.63 (76.94–88.76)	806
Vascular disorders	Hypotension	6.73 (6.17–7.35)	6.61 (2456.94)	6.59 (6.04–7.19)	516
Metabolism and nutrition disorders	Propofol infusion syndrome	7169.53 (5986.06–8586.99)	7028.09 (8,33,477.1)	1752.23 (1462.99–2098.66)	476
Cardiac disorders	Cardiac arrest	11.48 (10.36–12.72)	11.32 (3468.38)	11.27 (10.17–12.49)	370
General disorders and administration site conditions	Pyrexia	2.72 (2.45–3.02)	2.7 (390.9)	2.69 (2.43–2.99)	365
Immune system disorders	Anaphylactic reaction	15.58 (13.94–17.42)	15.39 (4241.38)	15.3 (13.69–17.1)	317
Musculoskeletal and connective tissue disorders	Rhabdomyolysis	18.59 (16.56–20.87)	18.38 (4795.94)	18.24 (16.25–20.47)	294
General disorders and administration site conditions	Chills	6.32 (5.63–7.1)	6.25 (1274.9)	6.24 (5.56–7.01)	289
Cardiac disorders	Bradycardia	11 (9.66–12.53)	10.91 (2061.92)	10.86 (9.54–12.37)	230
Respiratory, thoracic and mediastinal disorders	Bronchospasm	38.38 (33.5–43.97)	38.05 (7562.76)	37.46 (32.69–42.91)	213
Investigations	Oxygen saturation decreased	9.66 (8.41–11.1)	9.59 (1548.71)	9.55 (8.31–10.97)	202
Metabolism and nutrition disorders	Metabolic acidosis	16.72 (14.54–19.22)	16.59 (2910.55)	16.48 (14.33–18.95)	200
Cardiac disorders	Tachycardia	5.37 (4.65–6.21)	5.34 (651.7)	5.33 (4.61–6.16)	185
Investigations	Blood pressure decreased	6.35 (5.45–7.41)	6.32 (732.64)	6.3 (5.4–7.35)	164
Injury, poisoning and procedural complications	Medication error	7.13 (6.08–8.37)	7.09 (793.72)	7.07 (6.03–8.3)	152
Vascular disorders	Circulatory collapse	20.48 (17.31–24.23)	20.37 (2520.08)	20.2 (17.07–23.9)	138
Respiratory, thoracic and mediastinal disorders	Respiratory depression	28.36 (23.92–33.62)	28.2 (3500.83)	27.88 (23.52–33.05)	135
Investigations	Blood creatine phosphokinase increased	10.89 (9.15–12.96)	10.84 (1138.09)	10.79 (9.07–12.84)	128
Vascular disorders	Shock	14.72 (12.35–17.53)	14.64 (1605.03)	14.56 (12.22–17.34)	127
Respiratory, thoracic and mediastinal disorders	Respiratory arrest	10.85 (9.07–12.96)	10.8 (1079.85)	10.75 (8.99–12.85)	122

CI = confidence interval, χ^2^ = chi-squared, EBGM = empirical Bayesian geometric mean, EBGML = the lower limit of 95% confidence intervals of empirical Bayesian geometric mean, EBGMU = the upper limit of 95% confidence intervals of empirical Bayesian geometric mean, PRR = proportional reporting ratio, PT = preferred term, ROR = reporting odds ratio, SOC = system organ class.

**Table 7:** Top 20 adverse events associated with propofol in the elderly population, ranked by frequency.

SOC	PT	ROR (95% CI)	PRR (χ^2^)	EBGM (EBGML−EBGMU)	N
Immune system disorders	Anaphylactic shock	113.25 (97.74–131.22)	108.34 (19,677.52)	104.4 (90.1–120.96)	192
Vascular disorders	Hypotension	7.12 (6.04–8.4)	6.92 (741.04)	6.9 (5.85–8.14)	146
Cardiac disorders	Cardiac arrest	13.15 (10.75–16.09)	12.88 (1059.81)	12.83 (10.48–15.69)	97
Cardiac disorders	Bradycardia	11.28 (8.99–14.16)	11.1 (696.99)	11.06 (8.81–13.89)	76
Immune system disorders	Anaphylactic reaction	25.73 (20.26–32.67)	25.34 (1599.88)	25.12 (19.79–31.9)	69
General disorders and administration site conditions	Chills	5.78 (4.37–7.64)	5.72 (194.82)	5.71 (4.32–7.55)	50
Cardiac disorders	Cardiorespiratory arrest	11.26 (8.3–15.26)	11.16 (387.28)	11.12 (8.2–15.08)	42
Investigations	Blood pressure decreased	6.15 (4.54–8.34)	6.1 (179.09)	6.09 (4.49–8.26)	42
Respiratory, thoracic and mediastinal disorders	Bronchospasm	32.19 (23.25–44.58)	31.93 (1096.6)	31.59 (22.81–43.74)	37
Musculoskeletal and connective tissue disorders	Rhabdomyolysis	8.4 (6.05–11.67)	8.34 (232.13)	8.32 (5.99–11.55)	36
Injury, poisoning and procedural complications	Delayed recovery from anesthesia	487.65 (332.82–714.5)	484.21 (12,773.13)	413.88 (282.48–606.42)	31
Investigations	Oxygen saturation decreased	4.9 (3.38–7.11)	4.88 (86.31)	4.87 (3.36–7.07)	28
Metabolism and nutrition disorders	Metabolic acidosis	9.27 (6.35–13.54)	9.22 (197.36)	9.19 (6.29–13.43)	27
Metabolism and nutrition disorders	Propofol infusion syndrome	7435.27 (3583.38–15,427.67)	7391.21 (53,366.64)	2053.84 (989.83–4261.57)	26
Cardiac disorders	Tachycardia	13.82 (9.32–20.5)	13.75 (294.25)	13.69 (9.23–20.3)	25
Vascular disorders	Circulatory collapse	4.55 (3.07–6.75)	4.53 (68.82)	4.53 (3.05–6.71)	25
Cardiac disorders	Ventricular fibrillation	23.25 (15.54–34.78)	23.13 (504.12)	22.95 (15.34–34.33)	24
Cardiac disorders	Pulseless electrical activity	52.46 (34.38–80.06)	52.2 (1085.1)	51.28 (33.6–78.26)	22
Cardiac disorders	Torsade de pointes	25.79 (16.93–39.28)	25.66 (516.88)	25.44 (16.7–38.75)	22
Respiratory, thoracic and mediastinal disorders	Respiratory arrest	12.31 (8.09–18.74)	12.26 (226.59)	12.21 (8.02–18.58)	22

CI = confidence interval, χ^2^ = chi-squared, EBGM = empirical Bayesian geometric mean, EBGML = the lower limit of 95% confidence intervals of empirical Bayesian geometric mean, EBGMU = the upper limit of 95% confidence intervals of empirical Bayesian geometric mean, PRR = proportional reporting ratio, PT = preferred term, ROR = reporting odds ratio, SOC = system organ class.

**Table 8:** Top 20 adverse events associated with propofol in the general population, ranked by signal strength.

SOC	PT	ROR (95% CI)	PRR (χ^2^)	EBGM (EBGML−EBGMU)	N
Metabolism and nutrition disorders	Propofol infusion syndrome	7169.53 (5986.06–8586.99)	7028.09 (8,33,477.1)	1752.23 (1462.99–2098.66)	476
Investigations	Bispectral index decreased	874.93 (232.1–3298.19)	874.82 (1904.34)	636.51 (168.85–2399.41)	3
Injury, poisoning and procedural complications	Acute postoperative sialadenitis	388.87 (134.92–1120.86)	388.81 (1326.21)	333.41 (115.67–960.99)	4
Injury, poisoning and procedural complications	Airway complication of anesthesia	379.44 (261.11–551.41)	378.94 (10,376.69)	326.13 (224.42–473.93)	32
Injury, poisoning and procedural complications	Anaesthetic complication pulmonary	315.38 (162.93–610.49)	315.25 (2759.62)	277.84 (143.53–537.82)	10
Injury, poisoning and procedural complications	Premature recovery from anesthesia	311.1 (109.59–883.13)	311.05 (1090.77)	274.57 (96.72–779.44)	4
Injury, poisoning and procedural complications	Delayed recovery from anesthesia	276.21 (222.45–342.95)	275.16 (22,479.73)	246.23 (198.31–305.73)	92
Injury, poisoning and procedural complications	Inadequate aseptic technique in use of product	245.87 (168.74–358.24)	245.56 (6611.16)	222.27 (152.55–323.86)	30
Infections and infestations	Pantoea agglomerans infection	243.8 (111.91–531.13)	243.73 (1532.08)	220.77 (101.34–480.95)	7
Surgical and medical procedures	Procedure aborted	233.31 (71.2–764.55)	233.29 (630.79)	212.17 (64.75–695.26)	3
Eye disorders	Holmes-adie pupil	212.1 (65.05–691.64)	212.08 (577.73)	194.49 (59.64–634.2)	3
Investigations	End-tidal CO_2_ increased	205.87 (63.22–670.33)	205.84 (561.95)	189.23 (58.12–616.16)	3
Investigations	Skin test negative	202.92 (98.52–417.97)	202.86 (1478.35)	186.71 (90.65–384.57)	8
Injury, poisoning and procedural complications	Anaesthetic complication cardiac	191.26 (76.84–476.02)	191.22 (874.44)	176.81 (71.04–440.06)	5
Investigations	End-tidal CO_2_ decreased	179.47 (55.46–580.8)	179.45 (494.34)	166.7 (51.51–539.48)	3
General disorders and administration site conditions	Hyperthermia malignant	178.05 (144.79–218.96)	177.34 (15,807.37)	164.88 (134.08–202.77)	97
Product issues	Product contamination microbial	174.41 (136.76–222.42)	173.91 (11,199.32)	161.91 (126.96–206.48)	70
Investigations	Brain stem auditory evoked response abnormal	159.08 (49.39–512.35)	159.06 (441.12)	148.97 (46.25–479.79)	3
Investigations	Fibrinolysis	155.55 (56.53–428.02)	155.52 (575.74)	145.87 (53.01–401.38)	4
Investigations	Allergy test negative	149.35 (73.07–305.27)	149.3 (1107.59)	140.38 (68.68–286.94)	8

CI = confidence interval, χ^2^ = chi-squared, EBGM = empirical Bayesian geometric mean, EBGML = the lower limit of 95% confidence intervals of empirical Bayesian geometric mean, EBGMU = the upper limit of 95% confidence intervals of empirical Bayesian geometric mean, PRR = proportional reporting ratio, PT = preferred term, ROR = reporting odds ratio, SOC = system organ class.

**Table 9:** Top 20 adverse events associated with propofol in the elderly population, ranked by signal strength.

SOC	PT	ROR (95% CI)	PRR (χ^2^)	EBGM (EBGML−EBGMU)	N
Metabolism and nutrition disorders	Propofol infusion syndrome	7435.27 (3583.38–15,427.67)	7391.21 (53,366.64)	2053.84 (989.83–4261.57)	26
Investigations	Brain stem auditory evoked response abnormal	4267.07 (712.81–25,543.95)	4264.16 (5114.59)	1706.26 (285.03–10,214.19)	3
Surgical and medical procedures	Procedure aborted	609.58 (175.12–2121.96)	609.17 (1500.06)	501.84 (144.17–1746.91)	3
Injury, poisoning and procedural complications	Delayed recovery from anesthesia	487.65 (332.82–714.5)	484.21 (12,773.13)	413.88 (282.48–606.42)	31
Nervous system disorders	Postinjection delirium sedation syndrome	474.55 (213.08–1056.89)	473.8 (2830.8)	406.25 (182.41–904.78)	7
Injury, poisoning and procedural complications	Unwanted awareness during anesthesia	421.53 (147.44–1205.21)	421.15 (1460.28)	366.94 (128.34–1049.11)	4
Investigations	Allergy test negative	406.39 (121.17–1362.95)	406.11 (1060.8)	355.47 (105.99–1192.19)	3
Neoplasms benign, malignant and unspecified (incl cysts and polyps)	Phaeochromocytoma crisis	299.51 (106.85–839.55)	299.24 (1075.74)	270.84 (96.62–759.17)	4
Injury, poisoning and procedural complications	Airway complication of anesthesia	251 (77.06–817.55)	250.83 (685.98)	230.58 (70.79–751.02)	3
Cardiac disorders	Acute right ventricular failure	242.16 (87.21–672.41)	241.94 (884.5)	223.04 (80.32–619.33)	4
General disorders and administration site conditions	Hyperthermia malignant	188.29 (92.01–385.34)	187.95 (1395.41)	176.36 (86.18–360.91)	8
Investigations	Mean arterial pressure decreased	158.04 (49.4–505.65)	157.93 (443.19)	149.67 (46.78–478.88)	3
Social circumstances	Homicide	145.92 (53.4–398.73)	145.78 (547.11)	138.72 (50.77–379.07)	4
Respiratory, thoracic and mediastinal disorders	Idiopathic interstitial pneumonia	144.74 (63.7–328.9)	144.55 (813.95)	137.6 (60.56–312.67)	6
Skin and subcutaneous tissue disorders	Circumoral edema	137.13 (50.26–374.15)	137 (515.21)	130.75 (47.92–356.75)	4
Injury, poisoning and procedural complications	Vasoplegia syndrome	134.55 (80.1–226.03)	134.09 (1892.3)	128.1 (76.25–215.19)	15
Immune system disorders	Anaphylactic shock	113.25 (97.74–131.22)	108.34 (19,677.52)	104.4 (90.1–120.96)	192
Investigations	Allergy test positive	112.29 (35.41–356.09)	112.21 (318.12)	107.99 (34.05–342.46)	3
Immune system disorders	Anaphylactoid shock	93.62 (38.39–228.28)	93.51 (443.05)	90.57 (37.14–220.83)	5
Injury, poisoning and procedural complications	Procedural hypotension	74.58 (33.14–167.85)	74.48 (423.88)	72.61 (32.26–163.4)	6

CI = confidence interval, χ^2^ = chi-squared, EBGM = empirical Bayesian geometric mean, EBGML = the lower limit of 95% confidence intervals of empirical Bayesian geometric mean, EBGMU = the upper limit of 95% confidence intervals of empirical Bayesian geometric mean, PRR = proportional reporting ratio, PT = preferred term, ROR = reporting odds ratio, SOC = system organ class.

### 3.5. Visual comparative analysis of cardiac ADE signals

**Figure 6.**
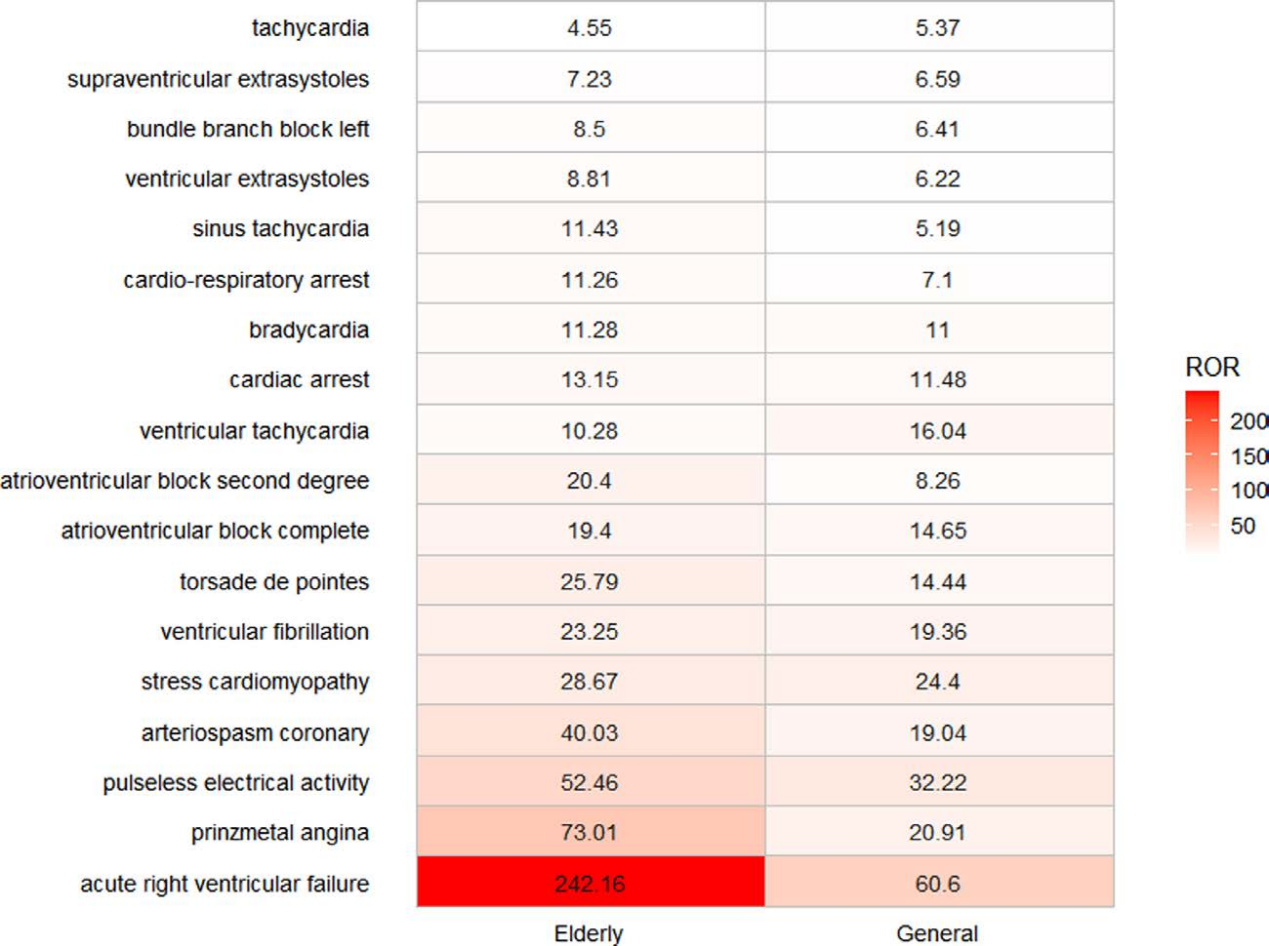
Heatmap of cardiac system organ class disorders. The color scale represents ROR values, ranging from 0 (white) to 250 (red), with darker colors indicating higher ROR values. ROR = reporting odds ratio.

To further characterize the cardiac safety profile of propofol in the general population versus the elderly, this study focused on PTs within the “cardiac disorders” SOC, using the ROR as the signal strength metric and color intensity to indicate signal magnitude (Fig. 6). Our findings demonstrate that acute right ventricular failure exhibited robust signals in both cohorts, with disproportionately higher reporting in the elderly population, thereby highlighting the necessity of age-sensitive pharmacovigilance and risk management in clinical practice. Conversely, tachycardia and ventricular tachycardia exhibited stronger signals in the general population than in the elderly. In contrast, most other cardiac-related adverse events showed stronger signals in the elderly cohort, including variant angina, pulseless electrical activity, coronary artery spasm, and stress cardiomyopathy. This may facilitate personalized therapy, optimize anesthetic agent selection, and enhance medication safety.

### 3.6. Comparator drug analysis result

**Figure 7.**
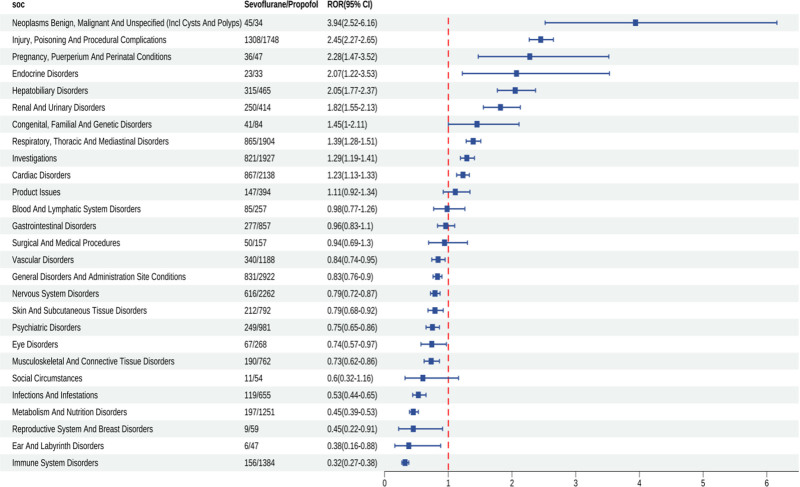
Sevoflurane versus propofol SOC level comparative analysis. CI = confidence interval, SOC = system organ class, ROR = reporting odds ratio.

**Figure 8.**
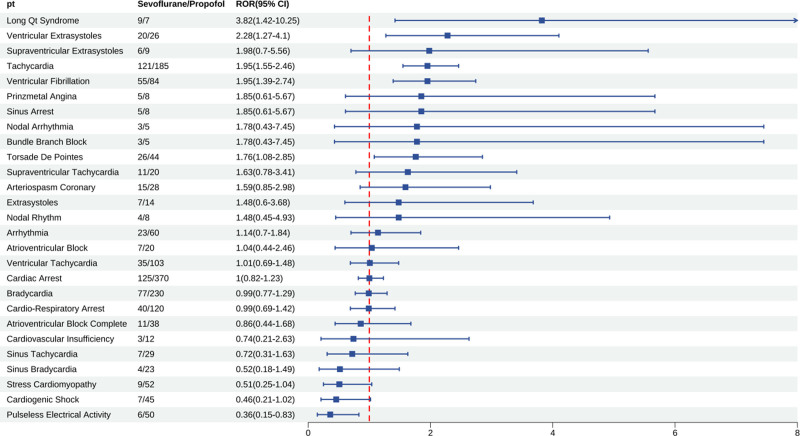
Sevofluranes versus propofol cardiac disorders PT level comparative analysis. CI = confidence interval, PT = preferred term, ROR = reporting odds ratio.

At the SOC level (Fig. 7), sevoflurane generated significantly stronger signals than propofol for hepatobiliary disorders, endocrine disorders, and renal and urinary disorders, and its cardiac disorders signal was also slightly higher, indicating that the prominent cardiac disorders signal is not propofol-specific. Conversely, sevoflurane showed markedly weaker signals than propofol for immune system disorders and metabolism and nutrition disorders. At the PT level within cardiac disorders (Fig. 8), 27 positive signals were shared by both agents. Sevoflurane produced markedly higher signals than propofol for long QT syndrome, ventricular extrasystoles, supraventricular extrasystoles, tachycardia, and ventricular fibrillation, whereas it was significantly lower for pulseless electrical activity, cardiogenic shock, stress cardiomyopathy, and sinus bradycardia. Notably, acute right ventricular failure was a propofol-specific signal and did not appear in the above comparison.

## 4. Discussion

Between 2004 and 2019, the annual number of propofol-related adverse event reports in the general population rose steadily, peaking at 547 cases in 2019. This upward trend likely reflects both the progressive extension of propofol’s market life and its expanding clinical use, which naturally increases the probability of AE reporting. After 2019, reports began to decline; although a modest rebound occurred from 2021 to 2024, volumes never surpassed the 2019 peak. This post-2019 decrease may be attributable to heightened prescriber awareness of propofol safety, more judicious clinical practices, and concurrent growth in market demand which could dilute the reporting rate. In the elderly subpopulation, the trajectory was more gradual but synchronous, mirroring the broader increase in surgical demand driven by population aging. Demographically, 53.1% of the general population reports involved female patients versus 46.9% male, possibly because propofol is more frequently administered in procedures that predominantly affect women, such as gynecologic surgeries. Conversely, among elderly patients, males predominated (53.0% vs 47.0%), a pattern that may stem from sex-specific physiology, differential surgical indications, altered pharmacokinetics, and clinical prescribing preferences in this age group.^[15]^ Geographically, the United States accounted for the largest share of reports (37.9%), consistent with its position as the primary propofol market and its robust pharmacovigilance infrastructure, which facilitates comprehensive and timely AE capture. Clinical outcomes underscored propofol’s inherent risks. In the general population, 10.3% of cases resulted in death, 21.5% were life-threatening, and 25.4% required hospitalization. Corresponding figures in the elderly were 9.7% death, 23.0% life-threatening events, and 29.0% hospitalization.

Both cohorts exhibited broad SOC involvement – 27 SOCs in the general population and 26 in the elderly. When the 2 sets of signals were compared, the elderly cohort generated positive signals in several clinically critical SOCs. These included: endocrine disorders, cardiac disorders, hepatobiliary disorders, immune system disorders, vascular and lymphatic disorders, gastrointestinal disorders, renal and urinary disorders. This disparity may arise from the amplification of true risk signals attributable to declining physiologic reserve capacity and multisystem organ dysfunction in elderly patients, amplification of propofol’s potential system-wide effects within the context of aging physiology. Future studies should integrate clinical data for further analysis.

The differences observed between sevoflurane and propofol at the cardiac disorder PT level are likely attributable to propofol’s characteristic profile of conduction block and right ventricular suppression. Acute right ventricular failure is not only the PT with the highest cardiac signal for propofol but also a propofol-specific signature, being significantly more pronounced in the elderly than in the overall population. Definitive conclusions will require future head-to-head randomized controlled trials or targeted trial emulation using real-world data.

In this study, PRIS exhibited extremely strong signals in both cohorts, suggesting a strong association with propofol use. PRIS – characterized by refractory arrhythmias, acute kidney injury, marked transaminase elevation, and potentially sudden cardiac arrest predominantly affects young children, critically ill geriatric patients, and those with neurological disorders, systemic inflammation, or severe infection.^[16,17]^ Although the exact pathogenesis of PRIS remains incompletely understood, studies have confirmed that its occurrence is positively correlated with propofol infusion rate, dose, and duration of continuous infusion.^[18]^ Therefore, strict control of infusion rate and duration is recommended in clinical practice, avoiding rates exceeding 5 mg/kg/h and duration beyond 48 hours. Prolonged infusion requires close monitoring of arterial pH, lactate, and creatine kinase levels.^[19]^ For patients who develop PRIS, the most supported intervention currently is immediate cessation of propofol infusion.^[20]^ Anaphylactic shock and hypotension were the most frequently reported PTs in both groups, with signal values marginally higher in the elderly. The anaphylactic potential of propofol is attributed to its phenol ring and isopropyl side chains acting as haptens that conjugate with plasma proteins to form complete antigens triggering IgE-mediated type-I hypersensitivity^[21]^; this relative risk is amplified in older adults whose immune competence is attenuated by thymic involution, reduced T-cell output, and impaired immunoregulatory cell function.^[22]^ Consequently, continuous monitoring of vital signs, oxygen saturation, and early allergic manifestations is obligatory during propofol administration to ensure prompt resuscitation if anaphylaxis occurs. Hypotension results from propofol-induced suppression of sympathetic tone and decreased systemic vascular resistance^[23]^; slow intravenous injection, preemptive volume loading, or coinduction with low-dose vasopressors effectively mitigate this adverse event.

Both the reporting rate and mortality of cardiovascular disease are high in the elderly population. Propofol exerts a dose-dependent depressive effect on right ventricular contractility.^[24]^ The corresponding signal for acute right ventricular failure was high in both cohorts, it was more pronounced in the elderly population, which exhibited higher reporting disproportionality. Conversely, tachycardia and ventricular tachycardia produced stronger signals in the general population, possibly because younger patients mount a more pronounced sympathetic response to propofol. In contrast, age-related down regulation of β-adrenergic receptors and degenerative changes in the cardiac conduction system^[25]^ predispose older patients to bradyarrhythmias such as sinus bradycardia or asystole. Across the cardiac disorder spectrum, the elderly exhibited significantly elevated ROR signals. Baseline cardiovascular disease interacts with propofol to yield more complicated clinical trajectories. Therefore, preoperative assessment of cardiac function should be meticulous; rapid bolus or high dose infusions must be avoided, the induction dose should be reduced by 40% to 50% to 1 to 1.5 mg/kg, and the maintenance dose reduced by 30% to 50%.^[26]^ Intraoperative monitoring should include continuous arterial pressure, ECG rhythm, and pulse oximetry, with vasoactive agents (e.g., norepinephrine or dopamine) readily available to maintain hemodynamic stability. For patients with severe underlying heart disease, alternative anesthetic strategies or combined regional techniques should be considered to reduce propofol exposure. Further studies are warranted to clarify the interplay between propofol-induced cardiotoxicity and age-related comorbidities.

Although this study employed multiple data mining approaches to dissect propofol-associated adverse events, its reliance on the spontaneous reporting FAERS database inevitably introduces inherent constraints that may compromise the accuracy and completeness of the findings. The database is subject to systematic biases such as underreporting, duplicate submissions, and incomplete case narratives, and overall data quality and integrity remain suboptimal. Propofol was launched at different times across countries, and this study exhibits a significant geographic imbalance in its sample distribution. Over 70% of the data originates from North America and Europe, with the United States alone accounting for 37.9% of the total sample size. In contrast, representation from Asia (excluding China, Japan, and South Korea), Africa, South America, and Oceania is severely underrepresented. Consequently, our findings predominantly reflect characteristics of European and American populations, and direct extrapolation to other geographic and ethnic groups globally should be approached with extreme caution. Future studies should prioritize establishing more diverse international cohorts to validate the generalizability of these observations. Simultaneously, systematic differences in the distribution of indications for ICU sedation versus surgical anesthesia may exist between the 2 cohorts. Future prospective multicenter real-world studies that integrate age-stratified population exposure data with clinical outcome information are warranted to determine whether these SOC level signals translate into quantifiable organ-specific risks. In parallel, mechanistic investigations using physiologically based pharmacokinetic modeling are recommended to elucidate how aging-related pathophysiological alterations modulate propofol’s organ-targeted toxicity, ultimately facilitating the development of predictive models and risk stratification strategies for personalized pharmacotherapy in geriatric patients. The crude age stratification that defines the elderly simply as ≥65 years obscures clinically relevant differences among the 65 to 74, 75 to 84, and ≥85 subgroups in physiological reserve, drug clearance, and comorbidity burden. Potential heterogeneity may exist and dilute the true risk in the extreme elderly population. This study employed 3 disproportionality algorithms (ROR, PRR, multi-item gamma Poisson shrinker) across 27 SOCs in 2 populations (general and elderly), yielding 162 statistical tests (3 × 27 × 2) without a formal multiplicity correction, which may result in an inflated probability of false-positive findings. Therefore, the results should be regarded as exploratory and hypothesis-generating rather than confirmatory. FAERS lacks structured exposure data. Our text mining cannot establish true dose–response relationships. The high sensitivity but low specificity of ROR and PRR predispose to false-positive signals, rather than causation or incidence.^[27]^ As no absolute risk estimates can be provided, the signals identified in our study are intended to quantify both absolute and relative risk to guide hypothesis generation; heightened vigilance and enhanced protective measures are therefore considered preventive strategies.

## 5. Conclusions

Based on the US FAERS, this study conducted mining and analysis of data on propofol-related ADEs from Q1 2004 to Q1 2025. The study identified potential age-related differences in adverse reactions and drug- specific signals for propofol. Owing to the inherent limitations of spontaneous reporting data, these findings should be interpreted as hypothesis-generating. The propofol-specific cardiac signals detected warrant prospective studies and randomized trials in the future, which could clarify exposure–response relationships, support individualized drug therapy, and help prevent adverse reactions.

## Author contributions

**Conceptualization:** Qirong Liu, Weijie Ma, Chouqi Hu.

**Data curation:** Qirong Liu, Weijie Ma, Chouqi Hu, Chunyue Fang.

**Formal analysis:** Qirong Liu, Chunyue Fang, Yuanyuan Zhong.

**Funding acquisition:** Qirong Liu, Yuanyuan Zhong, Jin Wen.

**Investigation:** Qirong Liu, Weijie Ma, Jin Wen.

**Methodology:** Qirong Liu, Weijie Ma, Chouqi Hu.

**Project administration:** Qirong Liu.

**Resources:** Qirong Liu, Weijie Ma.

**Software:** Qirong Liu, Weijie Ma, Chouqi Hu, Chunyue Fang, Jin Wen.

**Supervision:** Qirong Liu, Yuanyuan Zhong, Jin Wen.

**Validation:** Qirong Liu, Yuanyuan Zhong, Jin Wen.

**Visualization:** Qirong Liu, Chunyue Fang.

**Writing – original draft:** Qirong Liu.

**Writing – review & editing:** Qirong Liu, Jin Wen.
